# Community-Identified Implementation Strategies for Promoting the Adoption of HIV Self-Testing in a Southern California American Indian community: A Rapid Qualitative Analysis

**DOI:** 10.1007/s10461-024-04309-y

**Published:** 2024-04-09

**Authors:** Maximo R. Prescott, Jessica L. Montoya, Christina Perry, Ray Teran, Richard Armenta, Tommi L. Gaines

**Affiliations:** 1 San Diego State University/University of California San Diego Joint Doctoral Program in Clinical Psychology, San Diego, CA USA; 2 University of California, San Diego, La Jolla, CA USA; 3 Southern California Tribal Wellness Center, San Diego, CA USA; 4 California State University San Marcos, San Marcos, CA USA

**Keywords:** HIV, HIV testing, HIV prevention, HIV self-test, American Indian, Implementation science

## Abstract

HIV incidence increased by 18% between 2015 and 2019 among American Indians (AIs) despite declining rates in other racial/ethnic groups. Culturally-appropriate implementation of prevention programs is needed to address the intersectional conditions contributing to HIV vulnerabilities experienced by AIs. The objectives of this study were to understand factors influencing HIV testing decisions and identify implementation strategies to promote the acceptability of HIV self-testing (HIVST) in a southern California AI community. A total of 15 semi-structured interviews were completed with adult community members of a southern California AI reservation. Analysis used a rapid analytic approach that was guided by the Exploration, Preparation, Implementation, Sustainment (EPIS) framework and expert recommendations for implementing change (ERIC) compilation. Two team members applied a standardized summary template to elucidate implementation determinants and implementation strategies for adopting HIVST. Barriers to HIV testing identified by community members included HIV-related stigma and privacy concerns within their community. Community members expressed positive perceptions of the acceptability of HIVST, with many identifying ease of use and privacy as appealing attributes. Several implementation strategies were suggested for facilitating the adoption of HIVST, including increasing access to tests by mailing kits to community members and increasing demand for kits through media campaigns (e.g., local flyers, social media posts, and booths at tribal events). Community members also recommended discreet packaging of kits and cultural adaptation of accompanying materials (e.g., educational videos featuring community members). The qualitative input from AI community members facilitated identification of implementation strategies that may promote the acceptability and culturally-appropriateness of HIVST.

**Supplementary Information:**

The online version contains supplementary material available at 10.1007/s10461-024-04309-y.

## Resumen

Entre 2015 y 2019, la incidencia del VIH entre los indígenas norteamericanos (INs) aumentó un 18%, a pesar de que en otros grupos étnicos y raciales se observaron reducciones. La implementación culturalmente apropiada de programas preventivos es fundamental para abordar las circunstancias interseccionales que contribuyen a la vulnerabilidad al VIH entre INs. Los objetivos de este estudio fueron comprender los factores que influyen en la decisión de hacerse la prueba del VIH e identificar estrategias para fomentar la aceptabilidad de las auto pruebas, en una comunidad de INs en el Sur de California. Se realizaron 15 entrevistas semiestructuradas con los miembros adultos de una reserva situada en el Sur de California. El análisis se realizó utilizando un método cualitativo rápido, basado en el marco Exploration, Preparation, Implementation and Sustainment (EPIS) (español: Exploración, Preparación, Implementación y Sostenimiento) así como una recopilación de sugerencias de expertas en implementación de intervenciones, conocida como ERIC. Dos miembros del equipo de investigación utilizaron una plantilla estandarizada resumida para investigar factores determinantes y estrategias para la adopción de las auto pruebas del VIH. Los miembros de la reserva de INs identificaron el estigma asociado al VIH, así como preocupación por la privacidad entre la comunidad, como barreras para realizarse las pruebas del VIH. Los miembros reaccionaron positivamente a la aceptabilidad de las auto pruebas del VIH, destacándola facilidad del uso y la privacidad asociadas con estas pruebas. También, se sugirieron varias estrategias para facilitar la adopción de las auto pruebas, incluyendo el envío de pruebas a miembros de la comunidad y el aumento de la demanda de pruebas a través de difusión (e.g., folletos, publicaciones en las redes sociales, y puestos en eventos tribales). Los miembros también recomendaron que los empaques de las auto pruebas sean discretos, y que contengan materiales educativos culturalmente apropiados. Las contribuciones cualitativas de la comunidad INs ayudaron identificar estrategias de implementación que pueden fomentar la aceptabilidad de las auto pruebas del VIH en una manera culturalmente apropiada.

## Background

Although HIV incidence in the United States declined overall by 8% between 2015 and 2019, an alarming increase of 18% was observed among American Indian and Alaska Natives (AI/AN) [1]. HIV testing remains a crucial component of HIV prevention. Both the Centers for Disease Control and Prevention and the U.S. Preventative Task Force recommend universal HIV screening for all irrespective of risk [2] as awareness of HIV infection facilitates subsequent treatment, viral suppression, and decreased onward transmission of the virus [3]. Nearly 24% of AI/ANs with HIV are unaware of their HIV status compared to 16% of the general United States population [4], which suggests that AI/AN communities may experience greater barriers to accessing HIV testing than other groups. Previous research has found several factors that impact access to HIV testing and other HIV prevention services among AI communities, including limited availability of HIV prevention services, geographical remoteness of AI communities relative to the location of HIV prevention services, privacy concerns related to accessing HIV prevention services, HIV-related stigma, and low cultural competency among medical providers [5, 6].

HIV self-testing (HIVST) is a process through which an individual can privately collect, perform, and interpret the result of a HIV rapid diagnostic test in a location they choose. HIVST is a safe and highly effective alternative to clinic-based HIV testing as it has been found to improve testing frequency and uptake among communities disproportionately impacted by HIV (e.g., sexual and gender minorities [7], adults in Sub-Saharan Africa [8], and in resource-limited settings [8]). Although the potential of HIVST to improve access to HIV testing among individuals who may not otherwise test has been well demonstrated, the factors influencing successful implementation of HIVST in community settings are less understood.

Implementation science seeks to address this gap between the development of evidence-based practices (EBPs) and their real-world implementation in community and practice settings by studying the determinants (e.g., barriers and facilitators) of implementation and strategies for promoting the uptake of EBPs into use [9]. For this reason, implementation science frameworks, methodologies, and outcomes are considered critical to meeting the objectives of the ‘Ending the HIV Epidemic’ plan to reduce HIV incidence by 90% by 2030 [10]. Recent systematic review of implementation determinants and strategies for effective HIV interventions have found that research has primarily focused on characteristics of individual recipients of interventions and that HIV testing has received relatively less attention compared to pre-exposure prophylaxis (PrEP) in implementation science research [11, 12]. Furthermore, another systematic review of barriers and facilitators to HIV testing in migrant populations found a notable absence of HIVST studies within HIV testing implementation research [13]. Additional research is needed to better understand how implementation strategies for HIVST may be matched to determinants, particularly for understudied populations like AIs.

Previous studies among populations with risk factors for HIV infection have found HIVST to be acceptable due to its convenience and privacy [14], preferred to clinic-based HIV testing for stigmatized populations [15], appropriate, and feasible [16]. The successful uptake of HIVST likely requires community engagement [17], especially among AI communities that experience unique intersectional structural conditions that contribute to HIV vulnerabilities that stem from a history of colonization, discrimination, trauma, and violence.

Given the dearth of implementation science studies involving AI tribal communities to inform the implementation of HIV testing services, the objectives of the present qualitative study were to (1) understand factors influencing HIV testing decisions among AIs and (2) identify implementation strategies that may promote high acceptability of HIV self-testing in a southern California AI community.

## Methods

### Setting

The study population consisted of a Southern California AI reservation. The reservation is in a semi-rural region with a low population density and few healthcare services. Approximately half of the population within the reservation are under the age of 25 years and over half (53.4%) are uninsured. Furthermore, the reservation is in a county identified as having a high burden of HIV infections according to the U.S. Department of Health and Human Services [13]. Study activities were carried out in partnership with staff from a tribal wellness center located on the reservation. The wellness center was relatively new, launching a few months prior to the start of the current study. Research activities focused on the feasibility and acceptability of integrating HIVST within the tribal wellness center. Lastly, a community advisory board consisting of five tribal members and two tribal council members from the Tribes governing body oversaw the conduct of the study, providing input with recruitment activities, development of a qualitative interview guide, data interpretation and dissemination.

### Study Participants

A total of 15 community members from a southern California AI reservation were interviewed between July 2022 and November 2022. Individuals were eligible to participate if they were aged 18 years or older, affiliated with the tribal community (either work or lived on the reservation), and were able to speak English. Study participant recruitment was informed by a tribal steering committee and community advisory board formed for the study. Recruitment methods included setting up booths at tribal community events, posting study flyers around the community, mailing postcards with a study specific QR code, and receiving referrals to the study (e.g., word-of-mouth recruitment). The study was approved by the University of California, San Diego Institutional Review Board, and all participants provided informed consent prior to participation in the study procedures.

### Implementation Frameworks and Implementation Strategy Compilation

The design and analysis of the present study was informed by the Exploration, Preparation, Implementation, and Sustainment (EPIS) framework [18]. EPIS is both a process model that describes four phases of the implementation process and a determinant framework that posits several factors (i.e., characteristics of an evidence-based practice and outer and inner organizational contexts that are dynamic and interactive) that affect implementation of evidence-based practices. HIV prevention had already been identified as a public health priority based on previous community-engaged research [4] and HIVST as a potential evidence-based practice to help address the public health priority. The current study falls largely in the EPIS preparation phase of the implementation process, as our primary objectives were to identify implementation determinants of HIVST and implementation strategies to facilitate community uptake of HIVST. The inner context was conceptualized as the AI tribal wellness center, and the outer context as including and extending beyond the AI tribal community (e.g., state and federal levels of influence). The innovation factors were conceptualized as characteristics of HIVST that may impact its implementation and adoption. The final EPIS construct, referred to as bridging factors, represents factors that help link the inner and outer contexts, such as tribal-academic partnerships. Due to the specific reporting guidelines for the grant mechanism that funded the study, the study team was asked to present results post-hoc using the Consolidated Framework for Implementation Research (CFIR) [19]. A significant challenge in implementation science research has been inconsistency in terminology, which hampers systematic reviews of existing literature [20]. Thus, study results are presented in both EPIS and CFIR to improve the applicability of our findings to both frameworks and facilitate future synthesis of research.

The Expert Recommendations for Implementation Compilation (ERIC) was additionally used during analysis to categorize implementation strategies that participants recommended for promoting the adoption of HIVST within the AI community. The ERIC compilation is a taxonomy of 73 discrete implementation strategies that were identified using a modified Delphi process with a panel of expert panelists [21], which have subsequently been conceptually mapped into 9 broader categories of conceptually-related strategies [22]. The ERIC compilation provides a common nomenclature (i.e., implementation strategy terms, definitions, and categories) that ultimately facilitates the development of a multicomponent implementation strategy that can be tailored to the context of real-world settings to improve the adoption of EBPs, such as HIVST, into practice.

### Key Informant Interview Procedures

Key informant interviews were conducted by a trained research staff and utilized a semi-structured interview guide. The semi-structured interview guide was co-developed between the academic research team and the community advisory board. The guide included questions about general risk factors for HIV within the AI community, barriers to HIV testing, familiarity with the tribal wellness center, attitudes and beliefs about HIVST, and recommended strategies for implementing HIVST (See appendix for interview guide). These interview questions were developed based on the EPIS framework to elucidate what aspects of the outer setting (i.e., aspects of the community and its members), inner setting (i.e., familiarity with the tribal wellness program) and innovation factors (i.e., aspects of HIVST and its fit for the community) could potentially impact implementation and adoption. Based on feedback from the community advisory board, we included a brief discussion of STIs and HIV within the AI population prior to the start of the interview as this would provide background to ensure all community members had a basic understanding of both infections. During the interview, participants were shown a 5-min instructional video demonstrating the use of HIVST developed by the Center for Disease Control and Prevention. Participants were also shown an example of informational items that could be included with an HIVST kit, and this included condoms, a pre-exposure prophylaxis (PrEP) brochure, STI treatment guidelines, and a brochure for understanding HIV test results. Interviews were approximately 45 min in duration and conducted in person or virtually via videoconferencing. Of the 15 key informant interviews, 60% (n = 9) were conducted virtually via video conferencing and 40% (n = 6) were conducted in-person. Participants were compensated $50 for their participation. While a target of 25 key informant interviews had been initially been proposed, after 15 interviews the study team determined that saturation had been met as no new insights were emerging from interview summaries [23] during rapid analysis. Previous research has supported that qualitative studies can reach saturation within 9 to 17 interviews [24].

### Data Analysis

A rapid qualitative analysis [25, 26] was performed to quickly disseminate findings to the community advisory board and tribal leadership on a monthly basis during the duration of the research study and ultimately inform the implementation of HIVST. Rapid qualitative analysis has previously been shown to be more time efficient while maintaining the rigor and validity of traditional qualitative approaches [27–29]. Interviews were transcribed, verified, and de-identified prior to analysis. For rapid qualitative analysis, a standardized transcript summary guide based on a-priori domains from the interview guide and EPIS framework (i.e., these a-priori domains represented aspects of the inner, outer settings and innovation factors that could impact implementation) was first developed and piloted by two research team members before subsequently being applied to all interview transcripts. The first team member was responsible for summarizing all transcripts using the agreed upon template, which were subsequently reviewed by the second team member for accuracy. Furthermore, the piloting of the standardized transcript summary template and presentation of completed summaries were conducted with individuals from the authorship team and tribal steering committee (n = 7), which was diverse in both gender and age (between 18 – 65), to ensure validity. These summaries were transposed into a matrix table to allow for systematic analysis of both the breadth and depth of information elucidated by the qualitative data that was organized by a-priori domains following a matrix analysis [25, 26]. Informed by both the EPIS framework and ERIC compilation, two team members used the matrix method to identify the main themes pertaining to key implementation determinants and participant-identified implementation strategies for HIVST. Any disagreements in either summaries or themes were discussed and resolved by the two team members who conducted the qualitative analysis.

## Results

Community members identified numerous implementation determinants, which included barriers and facilitators to implementation of HIVST within their community. Implementation determinants were characterized using the EPIS framework, which guided the design and analysis of the study, but also mapped onto the constructs of the CFIR framework post-hoc for improved applicability (Table 1). The participant-identified implementation strategies were mapped onto implementation strategies defined by the ERIC compilation in Table 2.

**Table 1:** Implementation Determinants of HIVST in an American Indian Community Coded by EPIS and CFIR Frameworks

EPIS constructs	CFIR domain	CFIR construct	Theme	Exemplar quotes
Outer context: patient/client characteristics	Outer setting	Local attitudes	Low awareness about HIV/STIs, including relevance for AIs	I didn’t know that it [HIV] was increasing that much, … it kinda makes sense now that you say it, that it’s more popular in Native American reservations. So, I've never really ever heard of, like, a Native American person I know personally that ever—has ever, like, d—been deceased from HIV or anything like that… But most of the majority of the time, it's drug-related or it's COVID-related, 9 out of 10. It’s just scary. Like, it’s not surprising, I don’t think, honestly, just being from here. I’ve lived here all my whole life, and it’s not surprising, but it’s freakin’ scary, the—just the rates of like—like, it went down almost twice as much for other—like Asians.
Outer context: patient/client characteristics	Outer setting	Local attitudes	HIV-related stigma	I think the two stigmas of it is either that you’re a homosexual or either that you’re highly promiscuous and sexually irresponsible. And so, I think that, like I said, so no one wants to be necessarily categorized in any of those. …a lot of people gossip, and a lot of people talk and talk about other people's business in any small, tight community… there's a stigma around HIV that… you're having a lot of sex, a lot of sexual partners, or you are on drugs, or you use needles Like, sure, everyone wants to be healthy, but they don’t wanna get ridiculed for it, you know. And that’s the reality. If anyone knows that you’re gettin’ an HIV test, what people are gonna say, ‘Oh, you were bein’ nasty,’ or ‘You were with a nasty girl or a nasty guy,’ whichever the situation calls, right? …that’s the stigma. it'll be a stigma for them and their family and their generations after them and maybe reflection on their parents, that they didn't raise’em correctly, I really have never met, like, someone from the rez say, 'Hey, man, I got HIV,' or 'I got AIDS.' You know, like, I don't think anybody would tell me that… 'cause it's really personal… and people feel like, if they have any kind of disease, they feel like they're gonna be persecuted. Not like cancer. When somebody has cancer, people feel sorry for them
Outer context: patient/client characteristics	Outer setting	Local attitudes	Lack of privacy	And then the fear of like, well, I go to the clinic, so I’m like, ‘Everybody there knows me’…So I think it’d [HIV testing] be more private at home… I’ve gone to the clinic since I was little, so I’m like, everybody knows me there …everyone knows we got the clinic. And you could probably get your test there, whatever. But then the issue would be, do you trust the clinic to actually keep your stuff quiet? you don't wanna come up to someone [for a test] and it spreads, and the next thing you know you're, like, no one wants to talk to you 'cause you have such-and-such… I feel like there's, like, no, uh, safe place yet.
Inner context: individual characteristics	Individuals	Innovation recipients	Lack of familiarity/awareness	Mmm, not very [familiar], just because the Wellness Program on the reservation is just barely starting up. So, there's not really—I haven't used it and I'm not even sure if it's open yet. I, um, am not very familiar with it [tribal wellness center] at all… And so, this new wellness program is new… so I don’t know very much about it at all. I-I just went there for the first time like two weeks ago for a weigh-in.
			Holistic offerings	I’m just very excited about the [Center] and just doing those things, whether it’s, um, with HIV, or mental health, or, you know, physical health. Just ha—uh, optimizing wellness, um, for our community. So, I’m just excited knowing the numbers, that this is something that we’re giving attention to.
Innovation factor: innovation/EBP characteristics	Innovation	Innovation relative advantage Innovation complexity Innovation design	Straightforward, simple, easy	…very easy to use. If you tested…yourself at a at-home test with a COVID test, basically the same thing .. usable, user-friendly, um, pretty self-explanatory. Uh, and by doing that, like, it kinda makes it easy and, you know, comfortable. Um, and I don’t-I don’t see why it would be difficult for people to use it to get their results, or to test, or find out any-anything like that.
Innovation factor: innovation/EBP fit			Private/confidential	I think it's a great thing to have, and it's very confidential, and nobody has to know but you. … if you’re already testing yourself for HIV… you’re probably already very stressed, and I think that it’s just, like, one less thing to worry about is, like—just, uh, it [HIV Self-test] being so easy, and then being able to do it, like, at your own discretion at home. I think it’s amazing. I love it.
Innovation factor: innovation/EBP characteristics	Innovation	Innovation design	Privacy concerns	The hesitation would be the, ‘oh, if I'm seen with this, then everyone and their mom is gonna know that I think I might have something.’ But it's kind of like the same thing of being caught with a pregnancy test or caught buying one. It's like, ‘oh, shit. This is gonna be the newest gossip.’

**Table 2:** Community-engaged Implementation Strategies for HIVST with an American Indian Community

ERIC implementation strategy category	ERIC implementation strategies	Theme	Exemplar quotes
Adapt and tailor to context	Promote adaptability	Cultural adaptations	I think messaging to the community with people, um, that they identify with is very important… Not generic… with feathers and dream catchers … whether it’s local natives or whatnot, but something that’s more geared towards Native American, native country.
		Discreet packaging	If a tribal [center] doesn’t deliver it right and it says on the damn packaging that the people can see, oh, AIDS test or HIV, anything, anything at all. It doesn’t even matter if someone sees it. If you could see it, and they think someone might have seen it, they’re gonna say, ‘I don’t trust those guys. I don’t want nothin’ to do with it’
	Tailor strategies	Increase availability by mail	Some would be comfortable pickin’ it up at the center there… Other ones might ask you to mail it to’em in a discreet box, so it looks like a dang Amazon package or somethin’
Engage consumers	Use mass media	Increase HIVST awareness via flyers at tribal spaces, events, and social media	…flyers around the rec center or tribal hall. Um, social media is also really big, you know. Uh, and then also just-just, um, uh, just more, uh, more events that-that we-th—like we just had. You know, more events like that, health events, more awareness… I think would definitely, uh, help spread the word.
	Prepare patients/consumers to be active participants	Increase demand via HIV education	we need to start… taking care of ourselves… we talk about goin’ to…pow-wows, and gatherings, and sweat lodges to do self-care, but, shoot, you gotta do self-care, and making sure that you don’t have HIV… and other STDs, you know? And so, I think that it’s important that we know the numbers. Because that’s a big factor, knowing that. so just even getting that information out [HIV rates]… I believe and have confidence in our people that if you know there’s a problem, then you’ll address it… like with COVID, when we found out that COVID was affecting the Native American community more than any other community, then, you know, we were serious about it.

### Outer Context: AI Tribal Community

During interviews participants were generally surprised about the prevalence of HIV/STIs in AI communities. Although many community members acknowledged the health impact of substance use and COVID-19 in AI communities, few were aware of the increasing incidence of HIV and other STIs observed among AI. Additionally, several community members expressed either misperceptions or concerns about others’ misperceptions about HIV, including how HIV is transmitted and risk factors for transmission. Ultimately, low knowledge of HIV may act as a barrier to HIV testing as many individuals may not seek testing due to low perceived risk for HIV transmission. Relatedly, HIV-related stigma also emerged as a prominent barrier to HIV testing, such that participants expressed concern that seeking an HIV test would lead to others’ assuming things about their identities or behaviors (e.g., injection drug use, homosexuality, and/or sexual activity):

...a lot of people gossip, and a lot of people talk and talk about other people's business in any small, tight community... there's a stigma around HIV that... you're having a lot of sex, a lot of sexual partners, or you are on drugs, or you use needles

Given these concerns about HIV-related stigma, a lack of privacy emerged as an interrelated concern for accessing clinic-based HIV testing services. Although community members acknowledged that they had access to HIV testing currently through their local clinic, they perceived a lack of privacy due to the small and rural nature of the clinic where many community members are employed, which could deter testing. One participant described the importance of privacy during HIV testing:

you don't wanna come up to someone [for a test] and it spreads, and the next thing you know you're, like, no one wants to talk to you 'cause you have such-and-such... I feel like there's, like, no, uh, safe place yet.

### Inner Context: Tribal Wellness Center

At the time of the study interviews, the tribal wellness center had recently opened and was not widely known or used by many tribal members. However, the tribal wellness center was identified by the community advisory board as a potential tribal setting to distribute HIVST to the community. For this reason, community members had limited familiarity with the tribal wellness center. Among those who were aware of the tribal wellness center, community members expressed the importance of the tribal wellness center offering holistic programming that spans several health issues and health behaviors versus focusing on any specific health condition. Although familiarity was limited, community members expressed excitement about the new tribal wellness center and its purpose to promote wellness within their community:

I’m just very excited about the [Center] and just doing those things, whether it’s, um, with HIV, or mental health, or, you know, physical health. Just optimizing wellness, um, for our community. So, I’m just excited knowing the numbers, that this is something that we’re giving attention to.

### Innovation Factors: HIVST

Community members identified several features or qualities of HIVST that they perceived as acceptable and appropriate after watching a demonstration of HIVST and that may, in turn, facilitate its uptake among the community. Specifically, community members described the HIVST as acceptable because they found it to be straightforward, simple, and easy to use. One participant likened the ease of HIVST to using an at-home, self-administered COVID test:

...very easy to use. If you tested...yourself at-home test with a COVID test, [the HIVST is] basically the same thing

Given the prominent concerns about HIV-related stigma and the relative importance of privacy/confidentiality when accessing HIV testing, participants also expressed positive perceptions about the appropriateness of the intervention for their community. One participant described how the discretionary nature of HIVST could help facilitate its use:

... if you’re already testing yourself for HIV... you’re probably already very stressed, and I think that it’s just, like, one less thing to worry about is, like—just, uh, it [HIVST] being so easy, and then being able to do it, like, at your own discretion at home. I think it’s amazing. I love it.

Although community members reported that HIVST may facilitate HIV testing by mitigating interrelated concerns of privacy and stigma, some acknowledged that HIVST would not eliminate these barriers to HIV testing completely. The example quotation below suggests that privacy and stigma may still pose barriers to uptake of HIVST as the fear of judgment or presumed HIV-positive serostatus remains:

The hesitation would be the, ‘oh, if I'm seen with this, then everyone and their mom is gonna know that I think I might have something.’ But it's kind of like the same thing of being caught with a pregnancy test or caught buying one. It's like, ‘oh, shit. This is gonna be the newest gossip.’

### Community-Identified Implementation Strategies

Participants shared several recommended implementation strategies for supporting the use of HIVST within their community, several of which may help to mitigate the perceived barriers identified across the inner and outer settings. Broadly these community-engaged implementation strategies aim to engage consumers (the AI tribal community) and adapt/tailor to the unique context of an AI reservation community.

To engage consumers of HIVST and facilitate its implementation in settings within an AI reservation community (e.g., the tribal wellness center), many community members suggested that demand for HIVST could be increased by providing more education about HIV to the community. One individual shared how they believed their community would be more likely to engage in HIV prevention (e.g., testing for HIV) if they were more aware of its specific impact on AIs:

...so just even getting that information out [HIV rates] ... I believe and have confidence in our people that if you know there’s a problem, then you’ll address it... like with COVID, when we found out that COVID was affecting the Native American community more than any other community, then, you know, we were serious about it.

In addition to increasing the community’s awareness about HIV, participants also identified increasing the awareness about the availability of HIVST as important for successful implementation. Community members identified several methods for increasing awareness of the availability of HIVST through the tribal wellness center, including outreach at tribal events, newsletters, and advertisements through social media.

Community members also provided several examples of adaptations to the packaging and distribution of HIVST they believed would support its uptake within their community, including the resources provided with the HIVST kit and how individuals could access it. Community members suggested that HIVST be culturally adapted for AI, which included specific recommendations of featuring AIs on instructional and educational materials provided with the kit and packaging HIVST kits with material used for cultural healing practices (e.g., sage for smudging):

I think messaging to the community with people, um, that they identify with is very important... Not generic... with feathers and dream catchers ... whether it’s local natives or whatnot, but something that’s more geared towards Native American, native country.

Several community members also expressed the importance of using discreet packaging for HIVSTs, such that they would not be easily identifiable given their concerns about HIV stigma and a lack of privacy. Relatedly, community members often expressed interest in increasing the availability of HIVST by providing a mail delivery option in addition to offering pickup from the tribal wellness center:

Some would be comfortable pickin’ it up at the center there... Other ones might ask you to mail it to ’em in a discreet box, so it looks like a dang Amazon package or somethin’

## Discussion

### Principal Findings

HIVST has the potential to improve HIV testing uptake among communities that are disproportionately impacted by HIV, such as AI communities. This community-engaged research study aimed to identify implementation determinants of HIVST and implementation strategies to facilitate community uptake of HIVST. Community members from a Southern California AI reservation identified several barriers to HIV testing, including low awareness about the relevance of HIV within the AI population and interrelated concerns around HIV-related stigma and a perceived lack of privacy that would deter clinic-based HIV testing within their community. HIVST was perceived as acceptable and appropriate for the AI community largely due to its ease of use and mitigation of privacy and HIV-stigma related concerns. Furthermore, community members identified several potential implementation strategies to address these barriers and further improve the uptake of HIVST within their community, which included improving awareness about both HIV and HIVST and adapting the distribution and packaging of HIVST within the tribal community.

### Comparison with Prior Work

Previous research has found that low awareness and misperceptions of risk for HIV transmission are barriers to engagement with HIV prevention programs (including testing) among AI communities. In one study of urban AI/ANs, 44% of those reporting engagement in behaviors that confer risk for HIV transmission rated themselves as being at no or low risk for HIV infection and that higher perceived risk was independently associated with recent HIV testing [30]. AIs have previously reported that engagement with HIV prevention and testing may be improved if programming was culturally-tailored, incorporated traditional tribal practices, and leveraged cultural and community strengths [6, 31]. Randomized controlled trials have further demonstrated the importance of cultural adaptations for interventions targeting AIs, including: improvements in HIV/STI knowledge [32, 33], uptake of testing for other STIs, and reduced sexual risk taking [34]. Community members in the present study similarly recommended cultural adaptations to HIVST as an implementation strategy for improving its cultural appropriateness and ultimate uptake within the community. As a direct result of these recommendations, study researchers and tribal leadership collaborated to develop a promotional campaign on HIV testing that involved an educational video to support the implementation of HIVST kits within their reservation and normalize HIV testing within the community. The video features local AI community members and a tribal councilmember describing the importance of HIV testing and demonstrating use of HIVST. HIV-related stigma has previously been found to be a salient barrier to implementation of HIV prevention interventions in need of specific strategies to address the challenges it poses [11]. Our research suggests that multifaceted implementation strategies that include discrete strategies for engaging consumers and adapting/tailoring to context may help to improve the uptake of HIVST within AI communities by addressing HIV-related stigma.

Our findings suggest that the high acceptability and appropriateness of HIVST with an AI community was facilitated by the relative advantage of being able to test privately at home or another private location, which reduced concerns about HIV-related stigma when accessing HIV testing services. Other populations at high risk for HIV have similarly reported the option to test privately reduces stigma and encourages testing compared to in-clinic HIV testing [35]. HIVST was also found appealing due to its ease of use and convenience, which has similarly been found to be correlated with high acceptability among other populations such as racial and ethnic minority men who have sex with men [36]. Although community members believed that HIVST would mitigate some of the concerns about a lack of privacy and HIV-related stigma associated with clinic-based testing in a small semi-rural community, these concerns were not entirely eliminated. Several implementation strategies aimed at further adapting/tailoring to context, such as discreet packaging and delivery/mailing of kits, were identified by the community to further ensure that individuals could access HIVST while maintaining their privacy. Other marginalized populations, such as Black/African American and Hispanic/Latino MSM, have similarly reported a strong preference for accessing HIVST through the mail to mitigate privacy and transportation concerns [36], which have also been documented among AIs living in rural communities [5].

### Limitations and Strengths

This study has several limitations that should be considered when interpreting its findings. Firstly, given the small population size of the Tribal community we did not report potentially identifiable information (such as gender, age, education level) from community members to protect their identities. Although this limited our ability to examine potential differences based on demographic characteristics within this population, efforts were made to recruit a diversity of perspectives from the AI community. Relatedly, the nonprobability sampling of participants may be subject to self-selection bias as the decision to participate was up to participants and could impact the external validity of our findings. The limited purview of participants in the present study (i.e., community members) also largely limited our findings to implementation determinants and strategies aimed at the level of individual recipients. Future studies should consider incorporating a broader range of stakeholders (e.g., clinicians, tribal wellness program staff, tribal leadership) to help elucidate a multi-level understanding of determinants and strategies to promote the uptake of HIV testing with AI communities.

Despite these limitations, the study has strengths that are also important to consider. One such strength was the formation of a tribal-academic partnership to support the implementation of HIVST within an AI tribal community, which served as a bridging factor facilitating linkages between the inner (tribal wellness center) and outer contexts (the broader reservation community and local, state, and federal levels of influence). Bridging factors are often not considered and understudied despite their importance to facilitating implementation of evidence-based practices [37]. The novelty of our findings is also a significant strength, given the dearth of community-engaged research evaluating the acceptability and appropriateness of HIVST for AIs and identifying implementation strategies for promoting the uptake of HIVST.

## Conclusion

Qualitative interviews with members of a southern California AI community provided evidence of the acceptability and appropriateness of HIVST for this population, largely due to the perceived advantage of privacy and related mitigation of HIV-related stigma. HIVST may further be promoted by community-identified implementation strategies, such as cultural adaptations to instructional and educational materials provided with the kit and packaging HIVST kits with material used for cultural healing practices.

### Supplementary Information

Below is the link to the electronic supplementary material.

Supplementary file1 (DOCX 33 KB)
