# Supplementary material for: Community-Identified Implementation Strategies for Promoting the Adoption of HIV Self-Testing in a Southern California American Indian community: A Rapid Qualitative Analysis
Source: AIDS Behav. 2024 Apr 9;28(6):2101–12. doi: 10.1007/s10461-024-04309-y (PMC11161417; doi:10.1007/s10461-024-04309-y)
Supplement: Supplementary file 1 — Supplementary file1 (DOCX 33 KB) [file 10461_2024_4309_MOESM1_ESM.docx]

**Key Informant Interview Probe Sheet**

[Note: The following is a general outline of the topic areas that are expected to be covered, not the exact wording (or ordering) of how the questions will be asked.]

**NOTE: Interviewer can have camera on or off. **

Hello,

Is this **[name of participant]**? Good morning, how are you doing today? Before we get started do you need to use the restroom or get a glass of water before we get started?

I will now begin **recording.** Could I get a verbal confirmation from you that acknowledge I have begun recording?

| Interviewer Prompt:  Before we jump into the interview, I would like to share with you some information about HIV in Native American communities. In our experience, we learned it can be difficult to answer our questions without background info. We have learned that HIV is not always spoken about, so I apologize if you already know this information and find it boring, but we do not want to assume what people know. So, I would like to discuss why we think this study is important. PAUSE RECORDING, SHARE SCREEN of Two-Pager Background.  I’m about to give you a lot of information and give you time to reflect and pause. [Expectation they will be asked if need clarification after every paragraph make sure to pause] |
| --- |

**BEGIN RECORDING BEFORE PROCEEDING.** Alright, the recording has continued.

- *NOTE ANY REACTIONS/RESPONSES DURING THE PAUSES FROM THE BACKGROUND INFO:
  - During the pauses between sharing some background information on HIV, you expressed [surprise/appreciation/etc.] at [*information*]. May you explain a bit about why you were [surprised/interested/etc.]?
- Based on the information I shared, is there anything I can clarify, or are there any questions you have about HIV?
  - Was there any information that caught your attention or anything that was new to you? [which?]
  - What information would be helpful to share with community members?
  - Was there anything I can clarify, or are there any questions you have about HIV? (Happy to follow up with you at a later time on if there were remaining questions I could not answer)
  - What was your main takeaway?

Thank you for asking questions and wanting to learn some more about HIV. Now, we will shift a bit to ask about how communities view and understand HIV.

- Our goal is to protect the health of Native Americans. What are some things that might make [tribe] community members more vulnerable to getting HIV that would be important for us to know? [Probe by asking about behaviors that increases one’s chance of getting HIV.]
  - [Probe by asking about behaviors that increase one’s chance of getting HIV:
    - Access to health care, using drugs, sharing needles.]
  - [IF person does NOT want to speak for others]
    - What are all the way could guess on how they respond or might think about these things, we understand that this is just a guess.
    - Validate that what you are asking them is a difficult task
  - If mention Youth:
    - Can you tell me more about the youth? Do the [tribe] youth differ between other youth from reservations? (If mentioned)
    - Could you expand?
    - What do you mean by youth? Different age groups engaging in different behaviors?
  - What are things in your community that may have a higher chance of people getting HIV?
    - What can make more people lead to having HIV, in the way that people not washing their hands can lead to a person getting a cold.
  - May need to rephrase:
    - What we learned from the background info on Native people, the rates of HIV are not going down in the same way that it is going down for other races, if you had a guess of what’s going on, what would you think?
  - Explain vulnerable: What are things in the [tribe] community that might lead people to get HIV? What might increase the chance of getting HIV?
    - To review transmission: “Let’s start with what behavior you are familiar with that increases the chances of someone getting HIV. These include the modes of transmission I mentioned earlier – passed through blood, sex, during pregnancy and delivery. Of these, are there any that are going on in your community that make people have a higher chance of getting HIV?
  - If they specifically ask us how it’s transmitted:
    - HIV can be transmitted via the exchange of a variety of body fluids from infected people, such as blood, breast milk, semen, and vaginal secretions. HIV can also be transmitted from a mother to her child during pregnancy and delivery.” -WHO
- What gets in the way of people getting an HIV test on the reservation?
  - People who do not have doctors or speak with their doctor about HIV?
  - [IF person does NOT want to speak for others]
    - What are all the way could guess on how they respond or might think about these things, we understand that this is just a guess.
    - Validate that what you are asking them is a difficult task
  - If mentioned a clinic:
    - May you walk me through the steps you would take in accessing an HIV test at the clinic?
      - As you are going through, let me know where you feel your privacy might be violated, or where it can be protected.
    - If you feel concerned about your privacy at that clinic, has that changed whether you will seek care from there in the future?
      - If no longer seeking care, there: What are your options for seeking healthcare or health-related counseling at other locations?
      - What can be done to alleviate some of your concerns about privacy?
- What is your familiarity with the Tribal Wellness Program?
  - The Tribal Wellness Program has been around and provides programs on opioid use, overdose risk, and youth-directed programs.
  - How have you heard about this?
  - Why?
  - If mention a van/another separate part:
    - Where could you get more information about that van? May you please tell me more about the van you saw? What services were offered? Were people going up to the van? How often have you seen it/how often does it offer services?

| Interviewer Prompt:  Before continuing with the interview, we will pause and watch a short video describing an FDA approved HIV test that is self-administered and can be taken at home or other private location of one is choosing. After watching the video, I’ll ask you a few more questions. PAUSE RECORDING SHARE SCREEN, **SHARE SOUND**  <https://www.youtube.com/watch?v=6B73fe4U-iA> |
| --- |

**HIT RECORDING AFTER THE VIDEO:**

- Now that you’ve seen the video, what do you think of the HIV self-test?
  - Any thoughts you may have about it?
  - Did it seem like something people could use?
- Would people on the reservation be willing to take a HIV self-test? Why or why not?
  - What are the steps you would want someone to take if they were to get a positive HIV test?
  - It is similar to the COVID take home test
  - [IF person does NOT want to speak for others]
    - What are all the way could guess on how they respond or might think about these things, we understand that this is just a guess.
    - Validate that what you are asking them is a difficult task
- How might we be able to reduce some of the concerns people might have about HIV self-testing kits?
  - [Probe for reasons why they would be willing or would not be willing to use a HIV self-testing kit including concerns around stigma, lack of counselling before or after taking the test, linkage to care among those who test positive, costs, and privacy concerns].
  - If Stigma is mentioned:
    - How would you define stigma?
  - Based on previous answer
  - Walk us through your concern and how you would go about your privacy?
  - [IF person does NOT want to speak for others]
    - What are all the way could guess on how they respond or might think about these things, we understand that this is just a guess.
    - Validate that what you are asking them is a difficult task
- How do you think people on the reservation will want to get a HIV self-testing kit?
  - What is the best way to let people know about these options for getting a HIV self-testing kit?
  - [IF person does NOT want to speak for others]
    - What are all the way could guess on how they respond or might think about these things, we understand that this is just a guess.
    - Validate that what you are asking them is a difficult task
- What other information and resources should be included with the HIV self-testing kit?
  - I have an example of what could be included such as condoms, brochures on sexually transmitted diseases, prevention, and treatment options for HIV, etc.
  - [Share screen of HIV Self-Testing Box]
  - What would this information be helpful for?
- After taking an HIV test using the testing kit described in the video, what type of support should a person receive, regardless of whether the results are positive or negative?
  - [Probe about other services and resources to connect an individual to such as the Tribal Wellness Program, a clinic connected or not connected to [tribe], the [tribe] therapist on duty, a national hotline supported by federal agencies such as the CDC].
  - [IF person does NOT want to speak for others]
    - What are all the way could guess on how they respond or might think about these things, we understand that this is just a guess.
    - Validate that what you are asking them is a difficult task
  - Imagine someone getting a positive HIV test and imagine how they would feel. How can we as a community support them? What resources would they need?
    - “that is a heavy thought. Can you explain to me why you think your life might be over?” (Assess knowledge gap on living with HIV, changing life as they know it, shame)
  - On the flip, a person may have a negative test when they thought they were positive because of their recent behaviors. What do you think you would feel and would do afterwards?
  - What might be some resources to provide people who have a negative result?”
- Do you have any last remaining comments you would like to share before the end of our interview?

This concludes our interview.

-----------------End Prompt---------------------
